# Using Information Technology to Manage the COVID-19 Pandemic: Development of a Technical Framework Based on Practical Experience in China

**DOI:** 10.2196/19515

**Published:** 2020-06-08

**Authors:** Qing Ye, Jin Zhou, Hong Wu

**Affiliations:** 1 Tongji Hospital Tongji Medical College Huazhong University of Science and Technology Wuhan China; 2 School of Medicine and Health Management Tongji Medical College Huazhong University of Science and Technology Wuhan China

**Keywords:** COVID-19, pandemic, health informatics, health information technology, technical framework, privacy protection

## Abstract

**Background:**

The coronavirus disease (COVID-19) epidemic poses an enormous challenge to the global health system, and governments have taken active preventive and control measures. The health informatics community in China has actively taken action to leverage health information technologies for epidemic monitoring, detection, early warning, prevention and control, and other tasks.

**Objective:**

The aim of this study was to develop a technical framework to respond to the COVID-19 epidemic from a health informatics perspective.

**Methods:**

In this study, we collected health information technology–related information to understand the actions taken by the health informatics community in China during the COVID-19 outbreak and developed a health information technology framework for epidemic response based on health information technology–related measures and methods.

**Results:**

Based on the framework, we review specific health information technology practices for managing the outbreak in China, describe the highlights of their application in detail, and discuss critical issues to consider when using health information technology. Technologies employed include mobile and web-based services such as Internet hospitals and Wechat, big data analyses (including digital contact tracing through QR codes or epidemic prediction), cloud computing, Internet of things, Artificial Intelligence (including the use of drones, robots, and intelligent diagnoses), 5G telemedicine, and clinical information systems to facilitate clinical management for COVID-19.

**Conclusions:**

Practical experience in China shows that health information technologies play a pivotal role in responding to the COVID-19 epidemic.

## Introduction

The coronavirus disease (COVID-19) epidemic has taken on a global pandemic trend, posing a serious challenge to global health care systems [1]. During the outbreak of COVID-19 in Wuhan, China, the government enacted comprehensive and stringent preventive and control measures to bring the outbreak under control quickly. The health informatics community in China, including clinical informatics, public health informatics, consumer health informatics, and clinical research informatics, has actively taken action to leverage health information technology for epidemic monitoring, detection, early warning, prevention and control, and other tasks [2,3]. The Internet of Things (IoT) has provided platforms such as Worldometer [4] that enable people to access data to monitor the COVID-19 epidemic; integration of big data, such as transportation data and location-based services data, is used to model viral activity and provide guide for health care policy makers [5]; artificial intelligence (AI) and deep learning can enhance the detection and diagnosis of COVID-19 and facilitate the discovery of novel drugs [6]. Clearly, health information technology has played meritorious roles in the battle against COVID-19.

At present, as the COVID-19 epidemic has expanded worldwide, the task of epidemic prevention and control has become more arduous, and the world is facing enormous challenges. To provide theoretical and practical references to other counties on how health information technology can be used to respond to the COVID-19 epidemic as well as to various public health emergencies and disasters, in this study, we develop a technical framework responding to the COVID-19 epidemic from a health informatics perspective. Based on this framework, we also review specific health information technology practices for managing the outbreak in China, describe the highlights of these applications in detail, and finally discuss critical issues to consider when using health information technology.

## Methods

### Information Gathering and Framework Development

**Figure 1:**
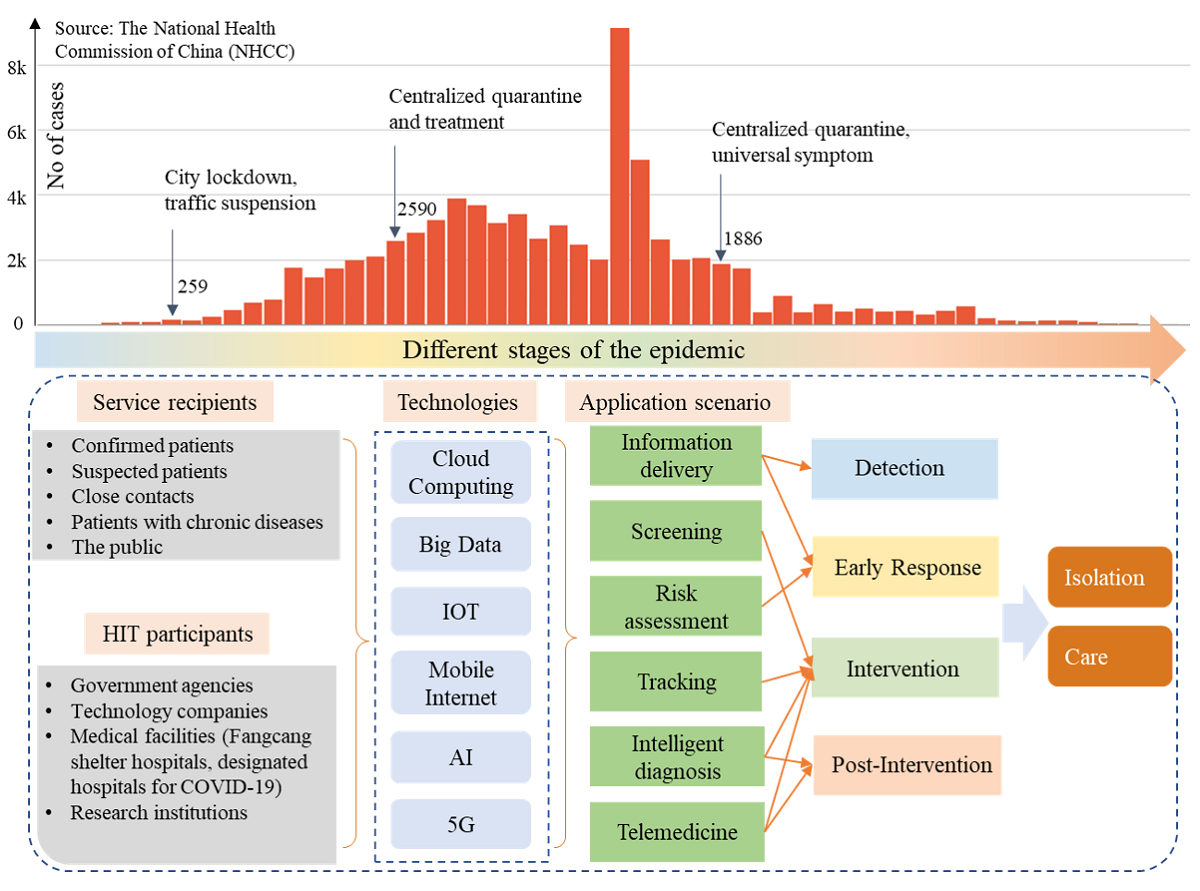
Proposed health information technology framework for responding to the COVID-19 epidemic. COVID-19: coronavirus disease. IoT: Internet of Things.

In this study, we collected health information technology–related information released by government departments and management agencies, medical institutions, health care industry associations, and public enterprises to understand the actions taken by the health informatics community in China during the COVID-19 outbreak and to develop a health information technology framework for epidemic response based on health information technology–related measures and methods (Figure 1).

We first defined and described the health information technology elements of response to an outbreak in terms of health information technology participants, service recipients, technologies, and application scenarios. Second, we constructed a complete technical response solution by correlating the health information technology elements with the different stages of the epidemic. Through this framework, we provide clear understanding of how health information technology is being applied to the epidemic response and how it functions at different stages of the epidemic.

The most effective measures to control infectious diseases are isolation and care; all activities to control the epidemic revolve around these two objectives, as has been demonstrated in China [7,8]. This framework includes health information technology participants, service recipients, technologies, and application scenarios around the four main stages of the COVID-19 epidemic: detection, early response, intervention, and postintervention.

### Definition of the Four Main Stages of the COVID-19 Epidemic

Based on the timeline of the epidemic in China, we identified four main stages (Figure 1): health information technology participants, service recipients, technologies, and application scenarios. This identification is consistent with previous studies [2].

#### Health Information Technology Participants

Health information technology participants include government agencies, technology companies, medical facilities (Fangcang shelter hospitals, which are designated hospitals for COVID-19), and research institutions, who leverage information technologies to respond to the epidemic in this national campaign [9], were identified.

#### Service Recipients

These include confirmed patients, suspected patients, close contacts, patients with chronic diseases, and the public [7,10,11].

#### Technologies

A variety of emerging technologies, such as cloud computing, big data, the IoT, mobile internet, AI, and fifth-generation mobile networks (5G), are being used for epidemic prevention and control. The transition of “5G+ Health” from the experimental to the clinical phase has been realized [12]. 

#### Application Scenarios

Application scenarios mainly include information delivery, case detection, screening, online services, risk assessment, and intelligent diagnosis. These scenarios are concrete manifestations of the integrated application of various information technologies for surveillance as well as prevention and control services [11,13-16].

## Results

### Health Information Technology Practice in China

Information technology has played a key role in China’s response to the COVID-19 outbreak. Information technology was used at all stages of the epidemic, such as prediction of epidemic trends, tracking of close contacts, and remote diagnosis. Based on the health information technology framework for responding to the epidemic described in the Methods section, we present specific health information technology practices for managing the COVID-19 outbreak in China (Table 1) and describe several health information technologies used to fight COVID-19 in detail.

**Table 1:** Health information technology practices for managing the COVID-19 outbreak in China.

Technology	Scenarios	Application	Examples	References
Mobile internet	Internet hospital, web-based services	Provide a variety of web-based services for the public during the outbreak, including screening and consultation services for mental health disorders or other diseases	Chunyu Yisheng, WeDoctor, China Mobile	Gong et al [11], Liu et al [13], Zheng et al [17], Sun et al [18]
	Web-based information dissemination platforms	Release official statistics about the COVID-19^a^ epidemic and keep the public correctly informed about the current situation in a timely fashion	People’s Daily, WeChat official account “Healthy China”	People’s Daily [19], NHCPRC [20]
Big data	Contact tracing	Record health status and activity trajectory, monitor crowd movement, or locate close contacts	Health QR codes, the Close Contact Detector app	Diao et al [21], Wang et al [22], Boulos et al [23], Ienca et al [2], Boulos et al [23]
	Epidemic prediction	Apply predictive modeling and turning point projection, monitor crowd activity	Predictive model for COVID-19	Wang et al [22], Liu et al [24]
	Spread track	Assist the development of epidemic prevention and control strategies	The dynamic information query system	Zhou et al [25], Peng et al [26]
Cloud computing	Supercomputing	Provide computing power	Supercomputing for big data analytics, vaccine development, and drug development	Ali Group [27], Liu et al [24], Li et al [28]
IoT^b^	Real time data collection	Intelligently manage information	Intelligent Diagnosis and Treatment Assistant Program	Bai et al [29]
AI^c^	Drones	Deploy for fever detection and crowd activity monitoring	DJI drones	Liu et al [24]
	Intelligent diagnosis	Assist doctors in CT^d^ diagnosis, reduce work pressure, and improve diagnostic accuracy	Deep learning–based computer-aided diagnostic system	Li et al [30], Gozes et al [15]
	Temperature detection	Rapidly measure body temperature	Airport infrared thermal cameras	Baidu [31]
	Robots	Use intelligent robots to perform simple operations such as disinfection and delivering medications and food during the epidemic	Robots for disinfection, delivering medications, and measuring vital signs	Brickwood [32], Huber [33], Yang et al [34]
5G	5G+ telemedicine	Provide support for remote video consultations and diagnostics	5G telehealth system	Paul [35], Augenstein [36], Li et al [37]
Comprehensive	Clinical information systems	Facilitate clinical management related to COVID-19	Electronic health records	Ren et al [38]

^a^COVID-19: coronavirus disease.

^b^IoT: Internet of Things.

^c^AI: artificial intelligence.

^d^CT: computed tomography.

### Internet Hospitals

At present, the global COVID-19 epidemic situation is very serious, and the task of epidemic prevention and control is highly challenging. In the early stages of the epidemic, fever clinics for outpatient and hospital beds were severely overloaded in some areas in China. Against this backdrop, local governments, health care institutions, and a range of companies in China are taking full advantage of mobile internet and 5G technologies to actively provide internet health care services by clinical experts from all over the country. Internet hospitals have played an important role in the prevention and control of epidemics in China [11].

During the COVID-19 outbreak, government agencies encouraged the provision of “Internet+” medical insurance services, which has significantly promoted the public utilization of Internet hospitals [39]. Due to the rapid increase in the number of confirmed cases and deaths during the epidemic, both medical staff and the public have experienced psychological problems, including anxiety and depression. As a result, internet hospitals have started to offer several types of online mental health services [40-42]. For patients with chronic diseases, home delivery services provided by internet hospitals are also in great demand during the outbreak.

### Health QR Codes

Health QR codes are a major innovation that have been used during the COVID-19 epidemic for individual tracking at the national level; this has played an important role in epidemic prevention and control as well as in enabling people to return to work [2,23]. People are required to show or scan their health QR code when entering and leaving public places such as communities, supermarkets, and subways. Therefore, a big data system can track the travel routines of an individual based on these records [23]. Health QR codes and big data technology can identify whether a member of the public has been in direct or indirect contact with a confirmed or suspected patient with COVID-19. Through traceability, government agencies can quickly locate potentially infected people and take timely measures to prevent the spread of the virus.

**Figure 2:**
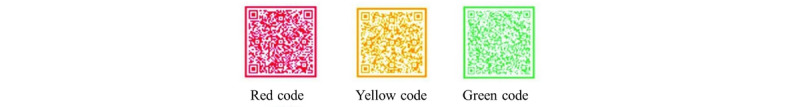
Health QR codes used in China during the COVID-19 epidemic. COVID-19: coronavirus disease.

There are currently three colors of health QR codes: red, yellow, and green (Figure 2). These colors indicate three states of health. Different regions have different definitions and requirements for red and yellow codes, while green codes uniformly indicate that a person currently has no symptoms of COVID-19; these codes can be used to quickly judge an individual’s health status. Health QR codes not only play a major role in epidemic prevention and control but will also greatly enhance the digital transformation of government and the efficiency of public services.

### Intelligent Diagnosis for Chest Computed Tomography Images

Early in the COVID-19 outbreak, researchers and the clinical informatics sector acted quickly to develop computer-assisted diagnostic products for COVID-19 in collaboration with radiologists [30]. These products have played an important role in screening patients for COVID-19; two of the reasons for this are outlined below.

First, real time reverse transcriptase–polymerase chain reaction (RT-PCR) test results of some patients will show false negative results; as a result, suspected or confirmed patients with COVID-19 are not detected, which is not conducive to disease prevention or control of the epidemic [43,44]. Chest computed tomography (CT) features combined with RT-PCR test results enable more reliable diagnosis in clinical practice. It has been suggested that in addition to RT-PCR results and epidemiological information, special attention should be paid to chest CT features and laboratory examination results [30]. Existing studies show that approximately 96% of patients with COVID-19 present with chest CT abnormalities; therefore, chest CT features are essential for identifying COVID-19 [30].

Second, although RT-PCR serves as the gold standard method for confirmation of COVID-19, the procedure takes a long time (approximately two hours); however, an AI-based diagnostic system can detect lesions of COVID-19 with high sensitivity within two minutes [45]. AI-based diagnosis systems accelerate the screening process for suspected patients, enable the triage of suspected patients in a shorter time, reduce the risk of cross-infection in health care facilities, and relieve the shortage of doctors during the COVID-19 epidemic.

### Critical Issues for the Health Informatics Community to Consider

#### Capabilities of Future Clinical Information Systems

During the COVID-19 epidemic, clinical informatics professionals have also actively participated in the provision of technical support for the admission and treatment of patients with COVID-19. China’s experience demonstrates that the design and development of future clinical information systems should focus on the following capabilities to better respond to public health emergencies.

The first capability is rapid deployment. The first three Fangcang shelter hospitals with 4000 beds in Wuhan were built in 29 hours [46]. Clinical information systems must be deployed within the same short time frame to support the admission and treatment of patients with mild to moderate COVID-19.

The second capability is information exchange [47]. In this outbreak, it has frequently been necessary to exchange information (eg, test results and patient referral information) between medical institutions, the Chinese Center for Disease Control and Prevention, the Fangcang shelter hospitals, and designated hospitals for patients with COVID-19. Information exchange capability should be the focus of future clinical information system design to respond to public health emergencies.

The third capability is rapid response of electronic health records (EHRs) to emergencies [3,38]. During this outbreak, clinical informatics professionals have configured the EHR to specifically respond to COVID-19. These configuration and adaptation measures include screen and triage processes, order tools, suspected case reports, and outbreak-related information statistics. A standardized EHR configuration process should be developed to quickly respond to public health emergencies.

#### Emerging Technologies for Public Health Emergencies

Unlike the severe acute respiratory syndrome (SARS) outbreak in 2003, the internet has become the main information platform during the COVID-19 outbreak, and the public can access dynamic information on the epidemic through various platforms. After nearly 20 years of development, China has made great strides in many emerging technology areas. In the fight against the COVID-19 epidemic, the Chinese government, medical institutions, and a range of technology companies have actively leveraged cloud computing, big data, the IoT, mobile internet, AI, blockchain, 5G, and other digital technologies to improve the efficiency of epidemic monitoring, virus tracking, disease prevention, control, and treatment, resource allocation, etc.

The public can access epidemic situation dynamics and prevention knowledge through the mobile internet. Big data technologies can be used for epidemic situation analysis, material allocation and monitoring of personnel movement. AI has been leveraged in intelligent diagnosis of medical imaging and temperature measurement technology based on computer vision and infrared technology. Telemedicine based on 5G technology has also played an important role in the treatment of patients with severe COVID-19 and in international cooperation in the battle against the outbreak. All these technologies are supported by cloud computing.

Through the Chinese experience, we can clearly see that emerging technologies have played very important roles in epidemic prevention and control; however, we should also note that substantial challenges remain to be faced. While many countries worldwide have well-developed surveillance and prevention systems in place, the health information technology community still has a lot of work to do in light of the global pandemic of COVID-19.

#### Privacy Protection

Tracing human activity is an important method of identifying the source of COVID-19 infection and controlling the spread of the virus. Individual health information based on health QR codes is an effective measure to monitor and limit the movement of people. In addition, the exchange of health information between different organizations and medical institutions is a basic requirement for guaranteeing patient treatment and care. Chinese practical experience has proven that under the premise of protecting personal privacy, the collection, reasonable use, and exchange of personal information can improve the efficiency and effectiveness of epidemic prevention and control. However, it is also required that personal information be used only with the consent of the person from whom it is collected and that it not be limited to key populations such as confirmed patients, suspected patients, and close contacts.

In the future, the health informatics community should consider establishing a unified framework for the exchange of epidemiological data, subject to privacy protection–related laws, to fully share information and facilitate the orderly flow of information between governments, agencies, and communities to address the challenges posed by public health emergencies and disasters [47].

## Discussion

This study makes important theoretical and practical contributions. First, this paper discusses informatics response and experience in responding to the COVID-19 epidemic in China through a health informatics lens. We may have made oversights in our case collection; however, that does not detract from our demonstration of the efforts made by the Chinese health information community and the results they have achieved. Practical experience in China has demonstrated that emerging technologies have unique advantages and can play pivotal roles in addressing major public health challenges. Other countries facing a COVID-19 pandemic should consider using health information technology as part of their public health response. The COVID-19 epidemic is a common challenge facing humanity, and as the epidemic spreads, health information professionals from all countries must share their experiences and work together to explore a complete information technology response framework to improve the response to the current COVID-19 pandemic and to future public health emergencies.

Second, future clinical information systems, personal health records, and big data infrastructures should be capable of rapid adaptation to public health emergencies, such as good interface design and information sharing. If large amounts of personal information are shared, care should be taken to protect user privacy. Moreover, we must consider multiple effects of information technology, such as the spread of “fake news” and rumors, as well as the ethical and privacy issues involved in the leverage of AI and big data technologies [48,49]. Due to the popularity of the Internet and social media, “information epidemics” often occur along with disease outbreaks; therefore, it is also very important to use information technology to increase the transparency of information on epidemics, reduce public panic, and enhance public confidence in the measures taken to combat epidemics [50,51]. During epidemic prevention and control periods, the technology side (technology companies) should prevent technology abuse, while the regulatory side (government agencies and platforms) should be careful to promote technology for the good and benefit of the public.

The COVID-19 epidemic is currently showing a global pandemic trend, and our understanding of the new coronavirus is deepening. Global health information technology practitioners should be proactive and use their professional skills to respond to the COVID-19 epidemic. Practice in China shows that health information technologies play a very important role in responding to the COVID-19 epidemic. Therefore, we believe that the health informatics communities in all countries should react quickly and make full use of health information technology to respond to the epidemic.

## Abbreviations

AI: artificial intelligence
COVID-19: coronavirus disease
CT: computerized tomography
EHR: electronic health record
IoT: Internet of Things
RT-PCR: reverse transcriptase–polymerase chain reaction
SARS: severe acute respiratory syndrome
